# Regulation of early gonocyte differentiation in zebrafish

**DOI:** 10.1042/BST20253046

**Published:** 2025-08-26

**Authors:** Miranda L. Wilson, Florence L. Marlow

**Affiliations:** Department of Cell, Developmental, and Regenerative Biology, Icahn School of Medicine at Mount Sinai, New York, NY, 10029, U.S.A

**Keywords:** bipotential gonad, gonadogenesis, indeterminant gonad, ovary, sexual differentiation, testis

## Abstract

Zebrafish have been and continue to be an important model organism for studies of fundamental biology and biomedicine, including reproductive development and the cell intrinsic and extrinsic mechanisms regulating early gonocyte differentiation. Wild zebrafish strains determine sex using a ZW genetic system wherein the maternally inherited sex chromosome determines the embryo’s sex. Like other species, including humans, regulation of conserved autosomal genes is crucial for gonocyte and sexual differentiation. How these conserved factors are regulated by the diverse mechanisms found throughout the animal kingdom is an active area of investigation. Domesticated zebrafish strains lack the ZW sex determination system found in wild strains and undergo gonocyte and sexual differentiation through a process exclusively governed by autosomal genes and nongenetic influences like environmental factors. Through mutational analysis, molecular genetics, and RNA sequencing, our understanding of the complexity of oocyte and spermatocyte differentiation has become clearer. In this review, we explore the most recent studies of the conserved and divergent mechanisms of gonocyte differentiation between wild and domesticated zebrafish as well as possible adaptations related to their domestication. Further, the contributions of individual genes and their molecular genetic hierarchy in regulating gonocyte differentiation are discussed and related to other species where relevant. We also address the recent characterization of a novel oocyte-progenitor and its potential implications in gonad differentiation. Finally, the role of gonocyte-extrinsic mechanisms, specifically communication between differentiating gonocytes and surrounding somatic gonad cells and the influence of resident and infiltrating immune cells, is discussed.

## Introduction

**Figure 1:**
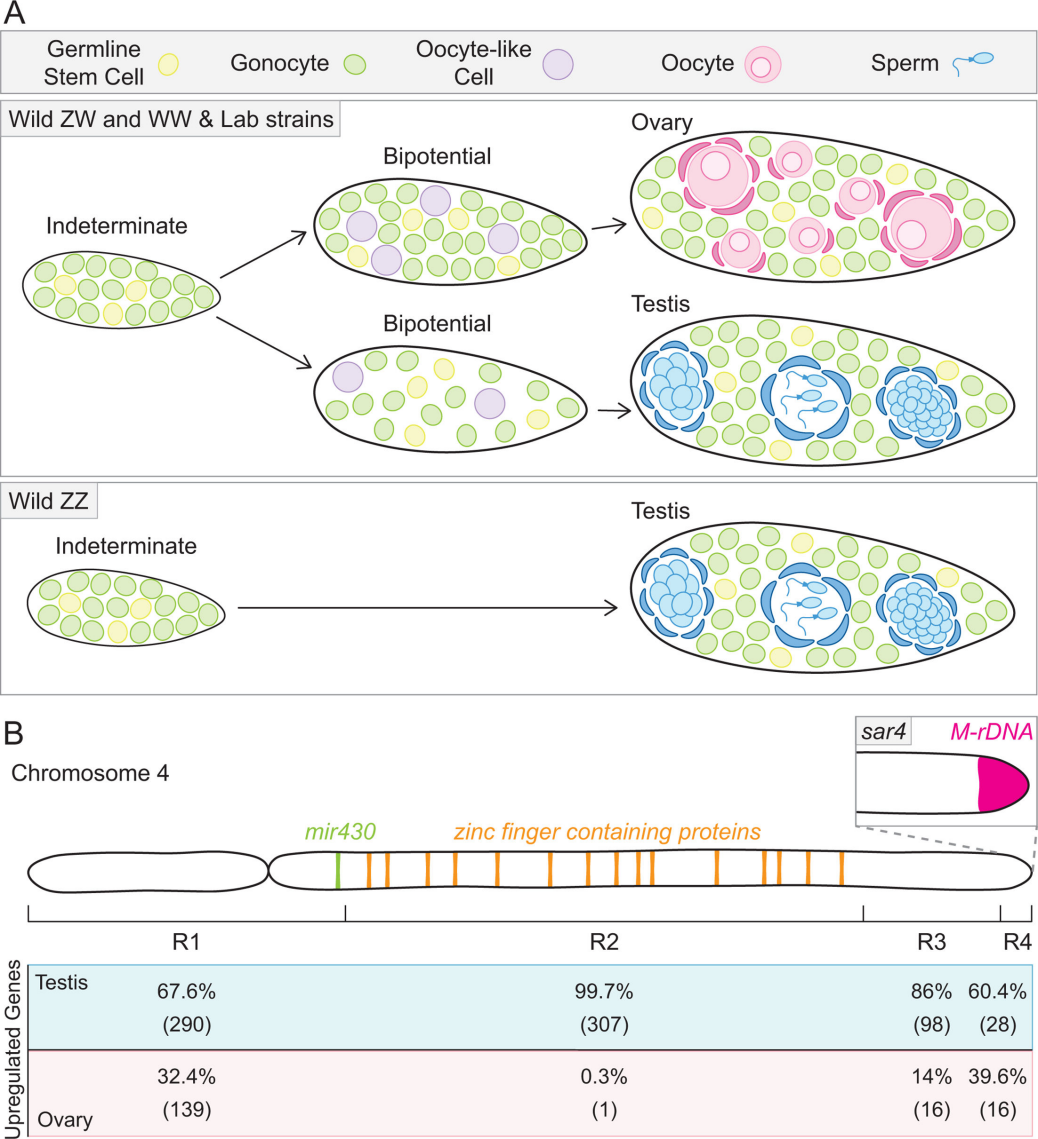
Gonadogenesis in wild and domesticated zebrafish. (**A**) Wild ZZ, ZW, WW, and domesticated laboratory zebrafish strains develop indeterminate gonads composed of gonocytes and germline stem cells. ZZ indeterminate gonads directly develop into testes, whereas wild ZW, WW, and lab strains establish a bipotential gonad containing oocyte-like cells [1]. The number of germ cells in the bipotential gonad has been linked to its developmental potential into either an ovary or testis, with fewer cells biasing to the latter [2]. Given the correct cues, the ZW, WW, and lab strain bipotential gonads can differentiate into either an ovary or testis. (**B**) Chromosome 4 has been partitioned into four transcriptionally distinct regions [3]. Overall, these regions are more highly expressed in testis, with region 2 being almost entirely transcriptionally silent in ovaries. Within region 4 of chromosome 4, the *sar4* locus contains an *rDNA* region termed *M-rDNA* that is highly expressed in ovaries [4]. Percentages represent the number of protein-coding genes expressed in that given region and the exact number of genes is indicated in parentheses. Gene expression data were compiled from [3].

Zebrafish is an important model organism for understanding the genetic basis of fertility and regulation of early gonad development. Domesticated lab strains lack a sex-determining chromosome, making them an excellent model for understanding the role of autosomal genes and external factors such as nutrition and rearing environment on gonad differentiation. Like most organisms, zebrafish undergo gonadogenesis through the differentiation of primordial germ cells into renewing germline stem cells (GSCs), which co-operate with somatic gonad endocrine cell precursors to establish a bipotential gonad composed of GSCs and gonocytes (Figure 1a) (reviewed in [5,6]). Gonocytes are mitotic cells that can differentiate into either oocytes or spermatocytes in response to specific cell intrinsic and extrinsic differentiation cues. Interestingly, if zebrafish gonocytes begin oocyte differentiation but conditions are not favorable for ovary development, the differentiating oocytes undergo apoptosis, allowing for testis development, and gonocytes instead differentiate into spermatocytes ([7], reviewed in [5,6]). Identifying the factors and mechanisms that drive the differentiation decisions of gonocytes in any species is a challenging endeavor. In domesticated zebrafish, this is even more complex as this process is polygenic and sensitive to external stimuli (reviewed in [5]). While major differentiation genes like *dmrt1* (*doublesex and mab-3 related transcription factor 1*), *cyp19a1a* (*cytochrome P450, family 19, subfamily A, polypeptide 1* a), and *sox9a* (*sry-box transcription factor 9* a) have conserved roles in gonocyte differentiation among vertebrates (reviewed in [5,6]), these factors do not comprise the entire repertoire of differentiation regulators nor the still elusive sex-determining trigger in zebrafish. This review compares and contrasts recent findings in studies of wild and domesticated zebrafish strains to highlight conserved aspects and adaptations of gonad differentiation between the strains. Additionally, recently characterized genes and cell extrinsic factors regulating early oocyte and/or spermatocyte differentiation will be explored and, where applicable, related to gonadogenesis mechanisms in other organisms.

## Chromosomal regulation of gonocyte differentiation in wildtype and domestic zebrafish

As mentioned, both wild and domesticated zebrafish form indeterminate gonads composed of plastic gonocytes that can differentiate into early oocytes or spermatocytes (Figure 1a). While some factors including *dmrt1* and *cyp19a1a* have conserved roles in vertebrate gonadogenesis, the field’s understanding of the mechanism(s) that promote oocyte and/or spermatocyte differentiation in zebrafish remains incomplete. Like other organisms, including all mammals, wild zebrafish determine their sex via a chromosome-based system. In contrast with the XX/XY system where the paternal chromosome determines sex, the maternal chromosome establishes sex in the ZZ/ZW system. Recent evidence indicates that the ZZ/ZW system was lost during domestication of laboratory strains [8]. Instead, laboratory strains undergo gonad differentiation using polygenic mechanisms [8–10]. Consequently, the key factors triggering oocyte versus spermatocyte development remain elusive. To address this, systematic characterization of gonad differentiation in wild zebrafish lines has been conducted to identify mechanisms that were maintained or diverged in domesticated fish.

A prevailing hypothesis in domesticated zebrafish is that indeterminate gonads become bipotential gonads with oocyte-like cells (OLCs) and are therefore more ‘ovary-like’ in character before sex determination and differentiation ([11], reviewed in [5,6]) (Figure 1a). Whether this developmental pattern is a shared feature of wild zebrafish or adapted during the domestication process was unclear. Wild zebrafish sex determination is controlled by a ZZ/ZW sex chromosome system (reviewed in [6]), wherein ZZ fish develop testes and ZW fish develop ovaries. Until recently, it was unclear whether dosage or factors exclusive to either chromosome directed ovary or testis development [1]. Using the Nadia wild strain, all ZZ indeterminate gonads were found to lack early OLC development and directly developed as testes [1] (Figure 1a). ZW and WW bipotential gonads, like laboratory strains, developed OLCs and were biased to ovary development, with occasional testis development [1] (Figure 1a). These findings indicate that factor(s) on the W locus are necessary for gonocyte development into oocytes and suggest that some domestic zebrafish lines retain this activity and may even be genetically WW. Further, wild Nadia and domestic wildtype Tüpfel long fin Z- and W- hybrids demonstrated that inheritance of the W locus correlated with elevated gonocyte numbers at ten days post fertilization [12], a phenotype strongly associated with subsequent ovary development [2] (Figure 1a). This is consistent with the developmental potential of ZW and WW bipotential gonads, wherein the presence of the W locus strongly biases to ovary differentiation but is permissive for testis differentiation [1]. Moreover, this finding is consistent with the wealth of data indicating that sex differentiation of domesticated strains is sensitive to external stimuli like nutritional availability and temperature (reviewed in [5]). However, in another domesticated strain (AB), a fraction of fish developed indeterminate gonads that directly develop as testes without forming an ‘ovary-like’ bipotential gonad, reminiscent of wild ZZ gonads [13]. These and other differences noted between domesticated zebrafish lines [9,14] could reflect distinct mechanistic adaptations developed during domestication.

Although the sex-determining trigger remains unclear, at least one autosomal locus appears to be conserved and similarly regulated between wild and domesticated zebrafish strains. The *sex-associated region* at the end of chromosome 4 (*sar4*) was first identified in wild strains [8] and is highly expressed in ovaries of the Nadia wild strain [3] and Tübingen/AB domestic strain [4] (Figure 1b). Notably, this region contains a ‘maternal’ ribosomal DNA locus (*M-rDNA*) that is highly amplified in domestic zebrafish ovaries [4,15] (Figure 1b) and is differentially methylated between males and females [4]. The sex-biased regulation of this locus could contribute to the function of *sar4* in gonocyte differentiation as high *M-rDNA* amplification correlates with low methylation in females [4]. Notably, ribosome biogenesis is an essential process in early zebrafish oogenesis [16]. Specifically, stage IB peri-nucleolar oocytes, which are enriched in nucleoli, the sites of ribosome biogenesis, are essential to oocyte and ovary development [17,18]. The association of ovary fate with the presence of numerous stage IB oocytes has led to a model wherein amplification and demethylation of the *M-rDNA* locus and consequent abundant *M-ribosomal RNA (rRNA*) expression in these cells may function as the elusive driver of ovary development and female differentiation. In alignment with this model, CRISPR/Cas9-mediated disruption of the *M-rDNA* locus revealed that higher mutation rates correlated with testis development, suggesting that higher *M-rRNA* transcription may be necessary for oocyte development [19]. Consistent with this, a recent study detected *M-rRNA* transcripts in adult zebrafish testes in varying amounts across samples [20], demonstrating that the *M-rRNA* expression is not isolated to oocytes and ovaries and supporting the hypothesis that ovary development may rely on a specific *M-rRNA* threshold rather than an all-or-nothing expression model. Further characterization is required to determine whether elevated expression from the *M-rDNA* locus is sufficient for oocyte development and to pinpoint the temporal window and aspect(s) of oocyte differentiation reliant on high *M-rRNA*.

In contrast with the ovary-biased expression of the *M-rDNA* locus, ‘Region 2′ of chromosome 4, a ~41.7 kb region adjacent to the *mir430* gene, is practically transcriptionally silent in both Nadia and AB ovaries [3] (Figure 1b). Thus, it appears regulation of this locus was maintained during domestication and its genes may alone, or in conjunction with other factors, be key to determining gonad sex and/or differentiation. Region 2 harbors a significant number of genes encoding zinc finger proteins [3] (Figure 1b), including some known to disrupt oogenesis [21,22] and spermatogenesis [23,24] in other systems. Therefore, these may be conserved regulators of gametogenesis. However, the majority of Region 2 genes remain unannotated with unknown functions [3] and deciphering the contributions of these genes to gonocyte differentiation is an exciting new avenue for further investigation.

## Meiotic machinery and meiosis entry

Meiosis is a germ cell-restricted process vital to generating genetic diversity and maintaining sexually reproducing species. Differentiation of gonocytes into early oocytes and spermatocytes coincides with the mitotic to meiotic transition. Following chromosome replication and condensation in leptotene of prophase I, homologous chromosomes pair via the synaptonemal complex, and the cohesion complex reinforces contact between paired chromosomes in zygotene. An enduring hypothesis in zebrafish gonad development is that ovarian differentiation is triggered by the entry and progression of oocytes through meiosis, which subsequently instructs the endocrine cell precursors of the somatic gonad to adopt ovarian cell fates (granulosa and theca) rather than testis-specific fates (Sertoli and Leydig) [25]. Although the presence of meiotic oocytes has been correlated with ovary differentiation in zebrafish, whether meiotic genes are systematically expressed earlier in female gonads as occurs in mammals remains to be determined. Furthermore, disruption or loss of synaptonemal and cohesion complex proteins has been linked to infertility in humans independent of sex (reviewed in [26,27]), thus understanding the role meiotic machinery and meiotic entry in early gonadogenesis is of high priority.

**Figure 2:**
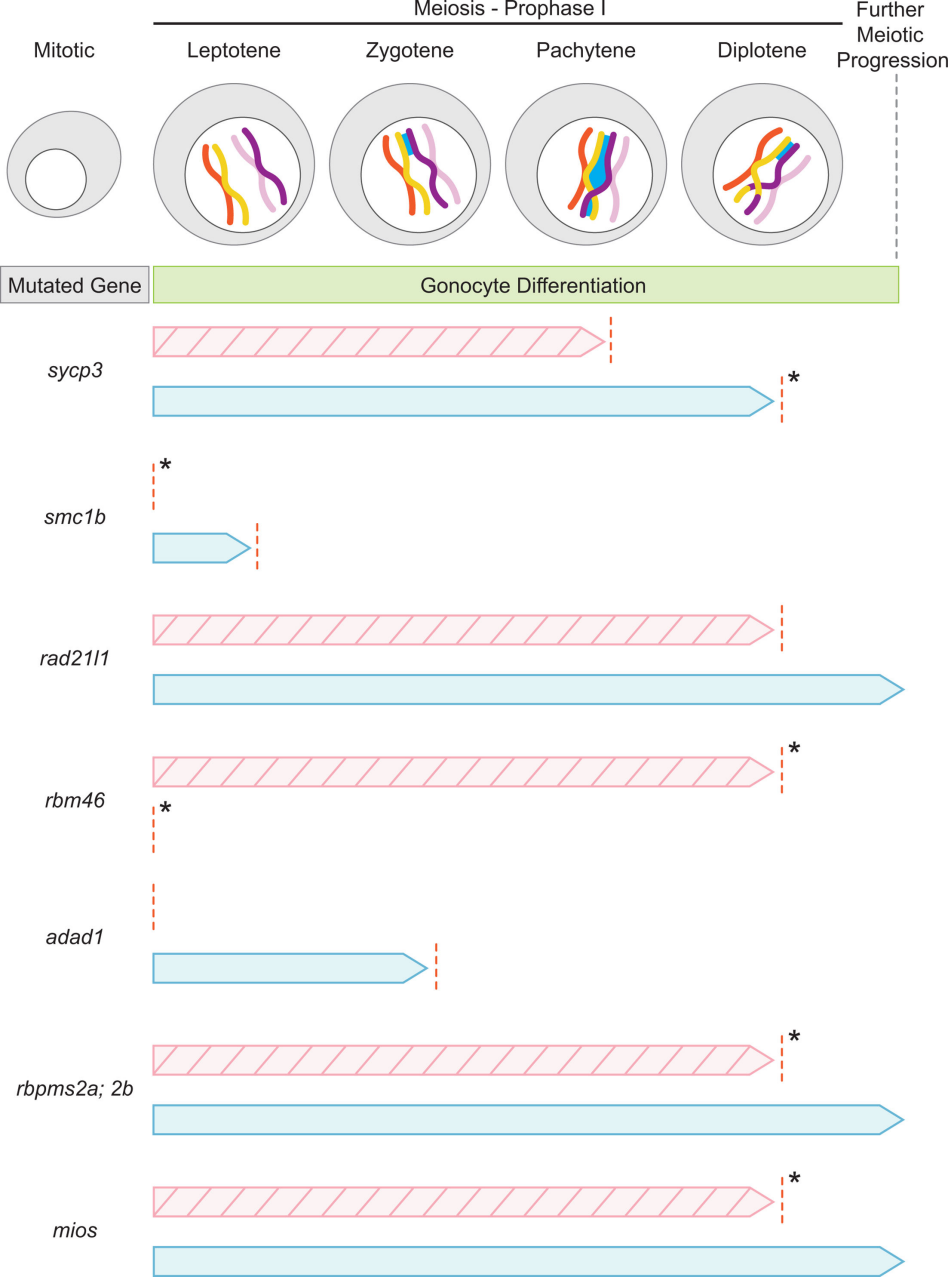
Meiotic progression defects in zebrafish mutants. Prophase I in zebrafish begins with leptotene, where sister chromatids condense and pair via cohesin proteins. The cells then progress from zygotene and initiate synaptonemal complex (dark blue) formation, which is fully intact and supports chromosome cross-overs in pachytene. In diplotene, chromosome cross-over is completed and the cells enter meiotic arrest [29]. The gonocyte differentiation trajectories of zebrafish mutant for the meiotic machinery and RNAbp genes discussed in this review are shown for oogenesis (striped, pink arrows) and spermatogenesis (light blue arrows). The arrows indicate the overall meiotic progression of mutant cells during prophase I, and the red dotted line indicates the most advanced stage of differentiation detected. The * indicates the inferred latest stage of meiosis I that the indicated mutant germ cells reach based on the available published data. RNAbp, RNA-binding protein.

In human testes, synaptonemal complex protein 3 (SYCP3) depletion strongly correlates with disruption of early spermatogenesis and infertility [28]. Similarly, zebrafish Sycp3 regulates gonocyte differentiation into both oocytes and spermatocytes [12]. All *sycp3^-/-^* fish are infertile males as neither mutant oocytes nor spermatocytes can progress through prophase I [12] (Figure 2). It is unclear whether oocyte apoptosis is triggered by abnormal synaptonemal complexes or persisting double-stranded breaks, but simultaneous loss of tumor protein (Tp53) suppresses oocyte loss [12]. Whether Sycp3 acts similarly in testes, has additional roles in oogenesis, or affects embryo ploidy remains to be determined as the fertility of *sycp3; tp53* mutants were not reported. This is especially pertinent because although mouse SYCP3 is dispensable for oogenesis, embryo viability is compromised by oocyte aneuploidy [30].

Structural maintenance of chromosomes 1B (Smc1b) is a cohesion complex component initially identified as a testis-specific protein in mice [31]. Zebrafish *smc1b* mutant germ cells have deficits in prophase I progression that cause all mutants to develop testes with spermatogenesis blocked at leptotene [32] (Figure 2). Histological analyses indicate no differences in the indeterminate gonad between control and *smc1b* mutants and suggest that *smc1b* mutants may directly develop testes without forming ‘ovary-like’ bipotential gonads [32] (Figure 2), establishing Smc1b as an essential regulator of the transition from gonocyte to early oocyte. Smc1b functions analogously in mouse gonadogenesis but appears to have a sexually dimorphic influence and may be more important for testis development [33]. Distinct *Smc1b* mutant allele phenotypes range from pachytene-blocked spermatogenesis to sterility, while mutant ovary phenotypes were milder, ranging from fewer oocytes with age to metaphase II arrest [33,34]. The differential sensitivity of gonadogenesis to Smc1b loss between species and alleles warrants further investigation into potential Smc1b involvement in human fertility disorders affecting early ovary development.

Like Smc1b in mouse, the cohesion complex protein Rad21 cohesin complex component like 1 (Rad21l1) appears to have sexually dimorphic roles in zebrafish oogenesis and spermatogenesis [35]. Its role in oocyte differentiation is essential as most mutant fish develop as fertile males, though a small proportion had germ cell deficits [35]. *rad21l1* mutant oocytes reach diplotene but are not maintained (Figure 2), suggesting Rad21l1, unlike Smc1b [32], is only required for later meiotic progression [35]. *rad21l1* mutation triggers activation of a Tp53 checkpoint, as loss of Tp53 was sufficient to restore oogenesis, albeit with impaired egg quality of *rad21l1; tp53* mutants likely due to persisting DSB abnormalities [35]. This observation and earlier studies of meiotic factors indicate that oogenesis but not embryogenesis can tolerate aneuploidy [25,36]. The role of Rad21l1 in gonadogenesis remains to be explored in other systems, including as a candidate for idiopathic human fertility disorders.

Retinoic acid (RA) signaling was long thought to be essential for meiotic initiation through STRA8 expression during mammalian oocyte and spermatocyte differentiation (reviewed in [37,38]). However, recent reports of normal oogenesis in female mice unable to synthesize [39] or respond to RA [40] and unperturbed spermatogenesis in male mice exposed to RA challenge this dogma [41]. RA contributions to zebrafish gonocyte differentiation have been unclear owing to lack of a Stra8 homolog [42]. The cellular RA-binding proteins (Crabp) function as intracellular transporters of RA, and zebrafish Crabp2a and Crabp2b are essential for early oogenesis [43]. *crabp2a;2b* mutants develop ‘ovary-like’ bipotential gonads with fewer early oocytes due to impaired germ cell proliferation, and, consequently, mutants almost exclusively form testes [43]. Based on the mutant meiotic entry and oocyte differentiation phenotypes, germ cell activation of a transgenic reporter for RA activity, and enrichment of endogenous Crabp2a protein in germ cells, the effect of Crabp2a and 2b on RA signaling in the developing gonad is proposed to be germ cell intrinsic [43]. These findings are similar with other teleost studies where the inhibition of RA synthesis delays meiotic entry in Nile tilapia and orange-spotted grouper [44,45], while loss of the RA-degrading enzyme Cyp26a1 induces earlier meiotic entry in both medaka and Nile tilapia [44,46]. The differences between mammalian and teleost responses to RA signaling during gonad differentiation may reflect the lack of a *stra8* homolog or differential use of Cyp26a1 versus Cyp26b1 (reviewed in [37]) as the RA-degrading enzyme. Identifying regulators of Crabp2-transduced RA signaling as well as potential RA-independent mechanisms in gonadogenesis is an exciting avenue for future studies and potential therapeutic intervention.

## RNA-binding proteins and meiotic progression

In addition to the meiosis regulators discussed, regulation of RNA metabolism is essential to gonocyte differentiation and progression through meiosis. RNA-binding proteins (RNAbp) are key regulators of RNA metabolism as they can regulate RNA transcription, translation, and stability, directly and indirectly. RNAbp functions in meiotic entry and progression and other crucial processes like ribosome biogenesis will be discussed. Further, RNAbps are known essential regulators of gonocyte differentiation in zebrafish (reviewed in [47]).

In zebrafish, RNA-binding motif protein 46 (Rbm46), a germ cell-specific RNAbp, regulates gonocyte differentiation into oocytes and spermatocytes [48]. *rbm46* mutants develop as infertile males with testes containing spermatogonia only (Figure 2), indicating Rbm46 controls meiotic progression [48]. *Rbm46* mutant mice contain ovaries devoid of oocytes [49] and testes with impaired spermatogenesis, suggesting Rbm46’s function in meiotic progression is conserved [49–51]. Further, lower expression of *Rbm46* may be causal for non-obstructive azoospermia in humans [50,52]. Histological analyses of *rbm46^-/-^* zebrafish gonads demonstrated an intact ‘ovary-like’ bipotential gonad and hallmarks of early oogenesis that were not maintained (Figure 2); thus, Rbm46 regulates meiotic progression in oocytes and spermatocytes [35]. Consistent with a lack of cells entering and progressing through meiosis, *rbm46* mutant gonads were deficient in RNA-encoding factors needed for progression through meiotic prophase I [48]. That simultaneous mutation of Tp53 failed to restore oocyte development in *rbm46^-/-^* fish [48] provides evidence that Rbm46 acts independent of Tp53 apoptotic regulation. Based on the studies in the mouse, RBM46 binds to several RNAs related to meiotic initiation and progression [49,51], including cohesins [51]. In the mouse, loss of RBM46 did not alter the abundance of cohesion-encoding RNAs in germ cells but instead affected overall protein levels, suggesting that RBM46 may regulate translation of its targets [51]. However, in another study, RBM46 knockout led to persisting expression of mitotic RNAs, positioning RBM46 as a key regulator of mitotic transcript abundance at the mitotic to meiotic transition [50]. Further, aberrant expression of human RBM46 is associated with male infertility. Thus, characterization of the RNA targets of Rbm46 and its contributions to posttranscriptional regulation of gene expression warrants further investigation [50].

Adenosine deaminase domain containing 1 (Adad1), a member of the adenosine deaminase acting on RNAs (Adar) protein family, is an RNAbp with tissue-specific expression in the ovary and testis of zebrafish [53]. In mice, ADAD1 expression is restricted to the testis [54,55], and late spermatogenesis and fertility are impaired in mutants [55,56]. Loss of *adad1* in zebrafish causes more severe phenotypes, as all mutants are sterile males [53]. In contrast with *Adad1^-/-^* mice, *adad1^-/-^* zebrafish spermatocytes initiate meiosis prophase I but cannot progress into pachytene [53] (Figure 2). Further, *adad1* mutant fish fail to initiate oogenesis [53] (Figure 2). This indicates that initial spermatocyte differentiation in zebrafish is more reliant on Adad1 than in mice. Further, its role in early oocyte differentiation in zebrafish, but not mice, suggests that mice may utilize other factors to fulfill Adad1’s more general role in meiotic prophase I entry of gonocyte differentiation, regardless of eventual sex. The observation that GSCs were not maintained in *adad1^-/-^* testes suggests that Adad1 and/or feedback from developing spermatocytes is required for GSC maintenance in the zebrafish testis [53]. Further investigation into the mechanisms that maintain GSCs is needed to distinguish these possibilities and has important implications for reproductive health.

Rbpms2 (RNA-binding protein, mRNA processing factor 2 a and 2b) is a well-established RNAbp-regulating gonocyte to oocyte differentiation. Unlike Rbm46, Rbpms2 is dispensable for spermatogenesis, mutants develop ‘ovary-like’ bipotential gonads, and all mutants develop as fertile males [57]. Further, Rbpms2 has dual roles in oocyte differentiation by promoting translation of RNAs encoding factors needed for oocyte development and repressing RNAs encoding spermatogenesis regulators [16,58]. Recently, Rbpms2 has been linked to the positive regulation of mTorc1 signaling during oocyte differentiation through the Gator2 complex protein, Mios (missing oocyte, meiosis regulator) [16]. The absence of Mios protein in *rbpms2* mutant early oocytes revealed Rbpms2’s role as a positive regulator of Mios translation. Diminished mTorc1, a known ribosome biogenesis regulator [59], altered oocyte nucleolar architecture and inhibited progression to the meiotic prophase I arrest checkpoint in both *rbpms2* and *mios* mutant oocytes (Figure 2) [16]. These phenotypes suggest that both *rbpms2* and *mios* are important for ribosome biogenesis and oocyte differentiation via mTorc1 signaling, though Rbpms2 appears to support *rRNA* transcription while Mios may function later in *rRNA* processing [16]. The more severe phenotypes of *rbpms2* mutants suggest that it has additional roles in ribosome biogenesis and oocyte differentiation beyond regulation of Mios and mTorc1. Given that *rDNA* and *rRNA* are limiting for nucleolar development [60] and that the *M-rDNA* locus discussed earlier has been implicated in ovary development, it will be interesting to determine whether Rbpms2, directly or through its RNA targets, supports ribosome biogenesis in differentiating gonocytes by regulating *M-rDNA* transcription or acts downstream to promote female sex determination [4,15,19]. Understanding how the *M-rDNA* locus and other regions of chromosome 4 are regulated may reveal the elusive trigger of sexual differentiation in zebrafish.

## Identification of an oocyte progenitor-stage gonocyte

Medaka gonadal sex is determined by an XX/XY-based system (reviewed in [61]). Forkhead-box L2 like (FoxL2l, formerly FoxL3) is a master regulator of oogenesis that is detected in mitotic germ cells that undergo a ‘gamete-committed’ division into meiotic cells [62]. That FoxL2l + germ cell numbers increase in XX fish but are lost in XY fish, and genetic evidence that FoxL2l loss does not affect spermatogenesis in XY fish while XX germ cells differentiate as sperm rather than oocytes, positioned FoxL2l as an essential regulator of oogenesis in medaka and, interestingly, suggests that gonocytes adopt an oocyte progenitor state prior to meiotic commitment [62].

Similarly, the zebrafish FoxL2l ortholog (Foxl2l) is expressed in premeiotic germ cells in early ovaries [63]. The majority of *foxl2l*-expressing cells rarely expressed the GSC marker *nanos2* or the early-meiosis marker *rec8a* and are transcriptionally distinct from the GSC and meiotic entry cell states [64], suggesting that these cells represent a distinct cell state [64,65]. Genetic studies demonstrated that, as in medaka, *foxl2l* is essential for zebrafish oogenesis and dispensable for testis development [63–66]. Consistent with a role for *foxl2l*-expressing cells as an intermediate state between the GSC and meiotic cells, *foxl2l* expression was expressed in indeterminate gonads of wild ZW and ZZ gonads [64]. However, histological and gene expression analyses indicate that *foxl2l* mutant lab strains fail to form oocytes and instead develop spermatocytes directly, like wild ZZ gonads; thus, Foxl2l is important for differentiation to the bipotential state [64,65]. Like other oocyte differentiation mutants [18,57,67–69], loss of *tp53* did not restore oogenesis in *foxl2l* mutants [66]. Further, loss of *dmrt1* inhibited spermatogenesis in *foxl2l* mutants but failed to restore oogenesis [65], indicating that the *foxl2l* progenitor is required for oogenesis and meiotic entry of oocytes, even in the absence of Dmrt1. Importantly, although both ZW and ZZ indeterminate gonads contain *foxl2l* positive cells, they are only maintained in ZW ovaries [70]. This further supports the notion that *foxl2l* expressing cells are an essential oocyte progenitor pool for OLCs in wild ZW/WW and domesticated bipotential gonads. Determining Foxl2l’s targets and how *foxl2l* expressing cells are maintained in ovaries remain open questions.

## Gonocyte extrinsic mechanisms of further differentiation

Communication between germ and endocrine somatic cells of the gonad is essential to both differentiation, maintenance, and function of the tissue across an organism’s lifetime. The endocrine somatic cells of the gonad, theca, and granulosa in ovaries and Sertoli and Leydig in testes generate sex-specific hormones and provide the environment necessary for gamete development. Therefore, it’s not surprising that disrupting interactions between germ and gonadal somatic cells is incompatible with successful gametogenesis and fertility.

**Figure 3:**
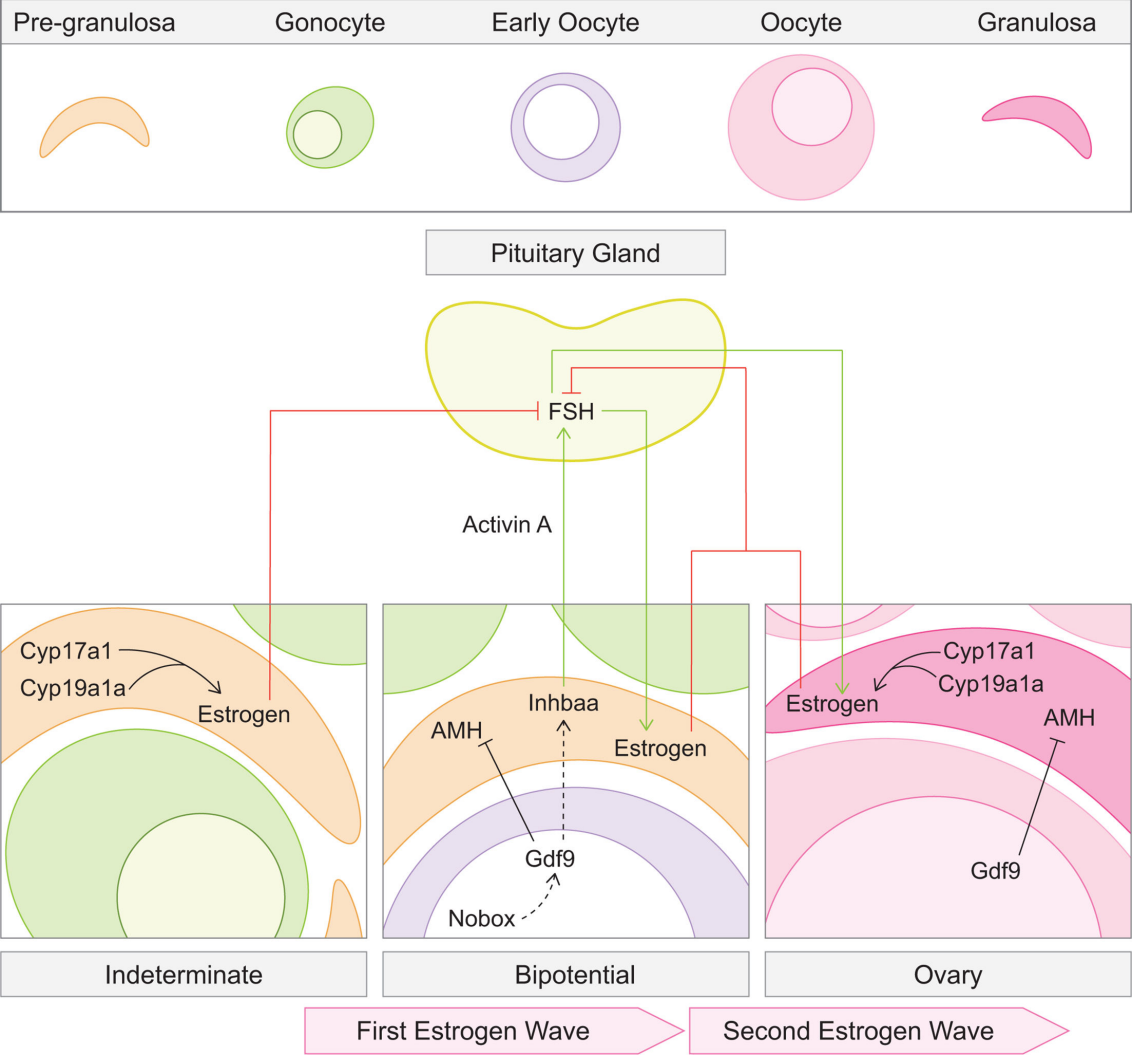
Estrogen synthesis and follicle maturation in oogenesis. In the indeterminate gonad, the precursor granulosa cells (orange) express Cyp17a1 and Cyp19a1a to initiate the first wave of estrogen synthesis in oogenesis. As the indeterminate gonad transitions into a bipotential gonad, Gdf9 in the early oocyte, likely positively regulated by Nobox, prompts expression of Inhbaa [76] by a currently unknown mechanism and inhibits anti-Müllerian hormone (AMH) production [77]. Inhbaa forms a homodimer, Activin A, which promotes follicle-stimulating hormone (FSH) expression by the pituitary gland to promote the second wave of estrogen synthesis. Upon ovary differentiation, the established granulosa cells (dark pink) express Cyp17a1 and Cyp19a1a to sustain estrogen synthesis while oocyte Gdf9 inhibits granulosa AMH production. FSH promotes estrogen synthesis and sufficient estrogen levels feedback to suppress further FSH production in mammals (reviewed in [78]). Based on the available genetic evidence, we hypothesize that this feedback loop is conserved in zebrafish.

Mutations disrupting Cyp17a1 (Cytochrome p450, family 17, subfamily A, polypeptide 1), an enzyme produced by theca cells to generate androgen that is later aromatized to estrogen by granulosa cells, are associated with infertility or sterility in humans and mice ([71], reviewed in [72]). In medaka, both XX and XY *sex-character-less* mutants (*cyp17a1* ortholog) undergo typical spermatogenesis but are infertile as they lack the sex-specific secondary characteristics and behaviors required for mating [73]. Similarly, *cyp17a1^-/-^* XX and XY Nile tilapia undergo complete spermatogenesis but are infertile due to 11 keto-testosterone deficiency [74]. As in medaka, zebrafish *cyp17a1^-/-^* differentiate as males with intact spermatogenesis but are infertile due to impaired mating behaviors [75]. The primary role of Cyp17a1 in zebrafish is estradiol production (Figure 3) as treatment with exogenous estradiol or testosterone, an estradiol precursor, restored oocyte development in *cyp17a1* mutants [75]. Further, like other mutants with impaired estrogen synthesis, including *cyp19a1a^-/-^* [58,79], loss of *dmrt1* in *cyp17a1* mutants can restore early oogenesis, but later oocyte stages are only achieved with exogenous estradiol [80]. Thus, distinct mechanisms regulate estrogen-dependent processes during ovary development (Figure 3) as development to the bipotential gonad stage can be compensated by elimination of Dmrt1, yet oocyte differentiation and follicle maturation cannot and are thus independent of Dmrt1 antagonism.

As in other organisms [81–84], the essential oocyte factor growth differentiation factor 9 (Gdf9) is expressed specifically in zebrafish oocytes [85]. Although zebrafish *gdf9* mutants initiate oogenesis, the oocytes fail to progress past the primary growth stage and consequently develop as fertile males [76]. Interestingly, *gdf9^-/-^* follicles are deficient in *inhibin subunit beta Aa* (*inhbaa*) [76]. In mouse granulosa cells, diminished *Inhba* (mouse ortholog) is associated with a ~38% decrease in fertility [86]. Zebrafish follicle cells, most likely granulosa cells as in mammals (reviewed in [87]), express *inhbaa* [88] which forms the Activin A homodimer responsible for follicle-stimulating hormone (FSH) production in the pituitary gland [89,90] (Figure 3). In mammals, decreasing estrogen levels stimulate FSH production to induce estradiol synthesis by granulosa cells ([91], reviewed in [92]). In zebrafish, rescue experiments place Gdf9 upstream of estrogen production because, like *cyp19a1a and cyp17a1* mutants [58,75,79,80], exogenous estradiol exposure prevents loss of mutant oocytes, but only through the primary growth stage [76]. Formation of the Inhibin A and B complexes that inhibit FSH production requires the Inhibin subunit alpha (Inha) protein (reviewed in [92]). Similar with exogenous estradiol treatment, removal of *inha* in *gdf9^-/-^* gonads restores *inhbaa* levels and improves follicle maturation but cannot support maturation [76]. Partial oogenesis restoration in these contexts suggests other Gdf9 targets are dysregulated. For example, Gdf9 is known to suppress anti-Müllerian hormone (AMH) production by granulosa cells to promote early zebrafish oogenesis [77]. Taken together, these findings indicate that a conserved role of Gdf9 is stimulation of Activin A production in granulosa cells to support the FSH and estradiol levels necessary for progression through oogenesis (Figure 3). How Gdf9 activity is controlled in zebrafish oocytes is unclear, but based on their similar mutant oogenesis defects, Nobox (*nobox oogenesis homeobox*) is a strong candidate (Figure 3) [93]. Regulation by NOBOX may be conserved as it also appears to regulate *Gdf9* expression in mouse oocytes [94].

Mounting evidence indicates that immune cells are not simply regulators of ovary and testis homeostasis and remodeling [95,96] but also influence gonadal differentiation ([97], reviewed in [98]). Forkhead box P3a (Foxp3a; FOXP3 in mammals) marks regulatory T (Treg) cells that modulate the immune responses of naïve and effector T cells and other inflammatory cells (reviewed in [99]). Mutation of *foxp3a* in zebrafish results in loss of *foxp3a+* Tregs in the gonad [100]. Histological analyses provided evidence that *foxp3a^-/-^* gonads initiate oogenesis but ultimately become sub-fertile males, and occasionally subfertile females [100]. Correspondingly, extensive apoptosis, elevated inflammatory factor RNAs, and inflammatory cell infiltration were observed in *foxp3a^-/-^* mutants’ testis [100]. Male and female infertility have been associated with inflammation in humans (reviewed in [101,102]), but the normal role of immune cells in gonadogenesis is not fully understood and is an important question, especially as immune contributions may vary depending on gonad sex [97].

**Perspectives**

- Highlight importance of the field: Deciphering the complex molecular genetic mechanisms influencing gonocyte differentiation is essential to understanding ovary and testis development and fertility. Further, characterizing the essential mechanisms regulating ovary and testis establishment in animal models has important translational implications for human fertility and understanding the etiology of reproductive conditions.
- Summary of the current thinking: Zebrafish gonocyte differentiation is influenced by a diverse milieu of cell intrinsic and extrinsic factors. Sexual differentiation is likely controlled by conserved and derived genetic mechanisms and may be influenced by the activities of tissue-resident and infiltrating immune cell populations.
- Comment on future directions: Further investigation into the developmental origins, differentiation, developmental potential, and contributions of the newly identified foxl2l^+ ^progenitor cell of theindeterminatet and ‘ovary-like’ bipotential gonad to fertility is needed. Additionally, understanding immune population dynamics in gonad development and maintenance is crucial for advancing diagnostics and treatments for fertility disorders.

## Abbreviations

GSCs: germline stem cells
OLCs: oocyte-like cells
RA: retinoic acid
RNAbp: RNA-binding protein
SYCP3: synaptonemal complex protein 3
